# BSA Nanoparticle Loaded Atorvastatin Calcium - A New Facet for an Old Drug

**DOI:** 10.1371/journal.pone.0086317

**Published:** 2014-02-03

**Authors:** Sripriyalakshmi. S, Anjali. C. H, George Priya Doss. C, Rajith B, Aswathy Ravindran

**Affiliations:** 1 Center for Nanotechnology and Advanced Biomaterials (CeNTAB), School of Chemical and Biotechnology, SASTRA University, Tirumalaisamudram, Thanjavur, India; 2 Medical Biotechnology Division, School of Biosciences and Technology, VIT University, Vellore, India; RMIT University, Australia

## Abstract

**Background:**

Currently, the discovery of effective chemotherapeutic agents poses a major challenge to the field of cancer biology. The present study focuses on enhancing the therapeutic and anti cancer properties of atorvastatin calcium loaded BSA (ATV-BSA) nanoparticles *in vitro*.

**Methodology/Results:**

BSA-ATV nanoparticles were prepared using desolvation technique. The process parameters were optimized based on the amount of desolvating agent, stabilization conditions as well as the concentration of the cross linker. The anti cancer properties of the protein coated ATV nanoparticles were tested on MiaPaCa-2 cell lines. *In vitro* release behavior of the drug from the carrier suggests that about 85% of the drug gets released after 72 hrs. Our studies show that ATV-BSA nanoparticles showed specific targeting and enhanced cytotoxicity to MiaPaCa-2 cells when compared to the bare ATV.

**Conclusion:**

We hereby propose that the possible mechanism of cellular uptake of albumin bound ATV could be through caveolin mediated endocytosis. Hence our studies open up new facet for an existing cholesterol drug as a potent anti-cancer agent.

## Introduction

Statins, the 3-hydroxy-3-methylglutaryl (HMG) – CoA reductase inhibitors, are among the commonly approved drugs to decrease the cholesterol level and prevent cardiovascular diseases. Some observational studies screened the effect of statins on the incidence of various cancers as part of the controlled trials on cardiovascular outcomes. Collectively, statins may play a dual crucial role as a lipid-lowering drug for the prevention of cardiovascular diseases and as an anticancer agent to prevent certain cancers. Of special interest is that the combination of statins with the traditional anticancer drugs like doxorubicin displayed enhanced anticancer properties of the lipid lowering agent [1]–[3]. This combinatorial therapy was able to surpass the multidrug resistance (MDR) in SH-SY5Y (human neuroblastoma) cells [4].

However, the poor solubility, low bioavailability (12%) and extensive hepatic first pass metabolism prevents it from being effective in most chemotherapeutic applications. These limitations can be overcome by conjugating the drug with appropriate excipients. Natural macromolecules have gained much interest as biomaterials owing to their inherent properties of biodegradability, lack of toxicity, and non antigenicity [5]. The well defined primary structure of albumin with the neighboring charged amino acid moieties allow the electrostatic adsorption of positively and negatively charged molecules on to its surface without the requirement of other compounds [6], [7]. In this regard, albumin nanoparticles has drawn significant attention in the clinical setting as a novel drug carrier owing to their high drug binding capacity and negligible side effects [8], [9]. Hence in lieu of adequate prior reports, the present study focuses on enhancing the therapeutic and anticancer properties of atorvastatin calcium loaded BSA (ATV-BSA) nanoparticles *in vitro* on MiaPaCa-2 cell lines.

## Materials and Methods

### Chemicals

Bovine serum albumin (BSA), ethanol and glutaraldehyde (25%) (desolvating agent and cross linking agent respectively), mannitol (cryo-protectant), MTS ((3-(4, 5-dimethylthiazol-2-yl)-5-(3-carboxymethoxyphenyl)-2-(4-sulfophenyl)-2H-tetrazolium)), were obtained from Sigma Aldrich, India. Atorvastatin calcium powder used for the experiments was of pharmaceutical grade. Dulbecco's Modified Eagle's Medium (DMEM) was supplied from Invitrogen. Propidium Iodide (PI) was purchased from Hi-Media chemicals; India. Double distilled de ionized water was used for all the experiments.

### Cell line

Human pancreatic cancer cell line (MiaPaCa-2) was obtained from National Centre for Cell Sciences (NCCS), Pune, India. The cells were grown in Dulbecco's modified Eagle's Medium (Invitrogen) supplemented with 10% (v/v) fetal bovine serum (FBS), 1% Penicillin-streptomycin and placed in an incubator with 5% CO_2_ at 37°C.

### Synthesis of BSA Nanoparticles

The synthesis of protein nanoparticles by desolvation method was done based on the method developed by Marty et al following minor modifications [10]. Briefly 0.1 g of the protein was taken and mixed with 2 mL of water. The pH was made to 8 followed by the addition of 8 mL ethanol drop wise at a rate of 0.8 ml/min under constant stirring. An opalescent solution was observed indicating the formation of the nanoparticles. Then 100 µl of the 8% glutaraldehyde solution was added for cross linking for increasing the stability of the particles. The solution was kept for stirring overnight to ensure the cross linking of all amino acid moieties. The stirred solution was then centrifuged at 15000 rpm for the nanoparticles to settle down. The supernatant was removed and the pellets were lyophilized using mannitol as cryoprotectant (Lyophilizer- Christ Alpha 2–4 Lo plus). Following lyophilization, the particles were suspended in the phosphate buffer saline (PBS) at pH 7.4 which resembles the physiological conditions in the body and prevented the denaturation of proteins.

### Synthesis of Drug Entrapped BSA Nanoparticles

Different concentrations of the drug was added to the protein solution and incubated overnight before the synthesis of the nanoparticles. Irache *et al* accounted on the sustained release of a drug when it was incubated with the protein prior to the formation of nanoparticles [11]. Incubation was followed by the production of the nanoparticles using desolvation process as mentioned above. The supernatant was collected after centrifugation at 15000 rpm. The coacervates obtained after centrifugation were lyophilised to obtain fine powder of the nanoformulation. Drug encapsulation efficiency was calculated by
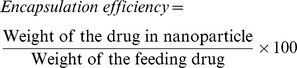



### Optimization of the process parameters

The various parameters that influence the synthesis of the BSA nanoparticles like pH, concentration of glutaraldehyde, and rate of addition of ethanol were studied and then optimized for the synthesis of the monodisperse particles with minimum particle size and maximum stability.

### Characterisation studies

#### Hydrodynamic size and Zeta potential

The mean particle size, zeta potential and the polydispersity index of the samples were determined using Photon Correlation Spectroscopy (PCS) using a Malvern Zeta sizer 3000 HS with He-Ne laser (633 nm) at a scattering angle of 90°C.

### Field emission Scanning Electron Microscopy (FE-SEM)

The morphological features of BSA nanoparticles before and after encapsulation were characterized by Scanning Electron Microscopy (JEOL JSM 670F-6701) at a voltage of around 3 keV.

#### FT-IR spectroscopy

FT-IR spectroscopy was performed in order to study the conformational changes on BSA owing to the interaction with the drug. Samples were ground with dry potassium bromide powder and compressed using a hydrostatic press. The spectra were recorded on a Perkin Elmer FT-IR spectrometer (Perkin Elmer, India) in a range of about 400–4000 cm^−1^.

#### Fluorescence spectroscopy

Fluorescence spectroscopy helps in understanding the residues that are involved in the drug-carrier interaction. The scanning was performed using Perkin Elmer fluorescence spectrometer (Perkin Elmer, India). The excitation wavelength was set as 293 and 280 nm to selectively excite tryptophan and tyrosine residues, and the emission was monitored in the range of 310–600 nm with the fixed slit width of 5 nm.

### Drug release kinetics

In order to estimate the release of the drug from the protein nanoparticles dialysis was done on ATV-BSA nanoparticles at pH 7.4 using PBS solution. The nanoformulation was placed in the dialysis bag in 5 mL PBS maintained at room temperature under constant stirring conditions. Equal volumes of PBS were withdrawn at predefined intervals of time, and the medium was replaced with the same volume of PBS. This study was carried out for about 80 hrs, and the concentration of drug released was estimated by determining the absorbance of the drug at 265 nm using a Perkin Elmer UV visible spectrometer (Lambda 25).

### *In vitro* studies

#### ROS assay

2′,7′-dichlorofluorescin diacetate (DCFH-DA) was used for the estimation of ROS production in the cells. Dichlorofluorescein, a highly fluorescent compound is obtained when DCFH-DA passively enters the cell and reacts with the ROS. Briefly MiaPaCa-2 cells were seeded in the 96 well plates and incubated for 24 hrs. The cells were then incubated with different concentrations of ATV-BSA and ATV for 48 hrs in order to monitor the cytotoxic activity of bare and protein encapsulated ATV. The media were removed and washed thrice with PBS. Approximately 150 µL of the reagent was added to the cells and kept at 37°C for 1 hr. After the removal of the reagent, fluorescence was determined at 485 nm excitation and 520 nm emission using a micro plate reader. The ROS level is expressed as the ratio of
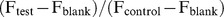
Where: F_test_ is the fluorescence intensity of the cells exposed to the nano particles.

F_control_ is the fluorescence intensity of the control cells.

F_blank_ is the fluorescence intensity of the wells without MiaPaCa-2 cells.

#### MTS assay

Cell viability was determined by 3-(4,5-dimethylthiazol-2-yl)-5-(3-carboxymethoxyphenyl)-2-(4-sulphophenyl)-2H-tetrazolium (MTS) dye-reduction assay which measures the mitochondrial respiratory function. Pancreatic cancer cell lines were seeded in 96 well plates and treated with test samples at different concentrations. MTS dye was then added to the cells and incubated at room temperature for 4 hrs. Absorbance was determined in a Tecan plate reader at 490 nm and was directly related to viable cell number.

The significant difference between the drug and nanoparticle encapsulated ATV were tested using independent T test.

#### Haemolysis assay

Haemolytic assay was done in order to study the haematocompatibility of the formulation. Blood was collected from healthy adult volunteers and loaded into test tubes containing EDTA (anti coagulant) and diluted with 0.9% saline. After that, 0.2 mL of diluted blood was added into fresh test tubes containing test samples and incubated for 60 min at room temperature. Similarly, 0.2 mL of diluted blood in 10 mL distilled water and 0.9% saline served as positive and negative controls respectively.

#### Cell Uptake studies

The cellular internalization of ATV-BSA nanoparticles by cancer cells were evaluated by fluorescent microscopy using propidium iodide staining. Propidium iodide is a polar dye and highly soluble in water [32]. For the cellular studies, the cells were seeded in a density of 10,000 cells/well in a 96 well plate. After 24 hrs of cell attachment, the wells were carefully washed with PBS followed by the addition of 100 µg/mL of ATV and ATV- BSA nanoparticles (in triplicates). After 24 hrs of incubation propidium iodide was added to each well and left for 15–20 min. The cells were washed with PBS and fixed in 5% paraformaldehyde followed by a final PBS wash. Finally, the samples were mounted on to glass slides with Distyrene Plasticizer Xylene (DPX) as the mountant and observed under a fluorescence microscope. The extent of dead cells obtained after treatment with bare ATV was compared with BSA entrapped ATV.

For confocal microscopic studies, ATV-BSA nanoparticles were labelled with fluorescein isothiocyanate (FITC) in 0.1 M carbonate buffer (pH 9) for 4 h at room temperature. Photo bleaching was avoided by keeping ATV-BSA-FITC conjugate under dark conditions. Later, DMEM medium was removed from the cells and washed with Dulbecco's Phosphate Buffered Saline, followed by the addition of ATV-BSA-FITC to the cells. This was then incubated for 2 hrs and the cover slip containing cells were kept on a laser scanning confocal microscope (FV1000, Olympus, Japan) to check the uptake of ATV-BSA-FITC conjugate inside the cells.

### *In silico* studies – molecular docking

Autodock4.0 suite was used to carry out the docking simulations. The crystal structure of BSA with PDB id 3V03 was retrieved from protein data bank (PDB) [12]. The structure of ligand atorvastatin was generated from smile strings followed by energy minimization. All the heteroatoms were removed. Hydrogen atoms were added to protein crystal structures using Autodock program while all non polar hydrogen atoms were merged. Lamarckian genetic algorithm which is based on adaptive local search was used as a search parameter. Short range van der Waals and electrostatic interactions, hydrogen bonding, entropy losses were included for energy based Autodock scoring function [13], [14]. The Lamarckian GA parameters used in the present study were numbers of run, 30; population size, 150; maximum number of evil; 25,000,000, number of generation; 27,000, rate of gene mutation; 0.02 and rate of cross over; 0.8. Docking was carried out using grid size 60, 60 and 60 along the X, Y and Z-axes with 0.375°A spacing. RMS cluster tolerance was set to 2.0°A. After the molecular docking, the ligand-receptor complexes were further analysed by using LIGPLOT [15] and Pymol [16].

## Results and Discussion

### Influence of the process parameters on nanoparticle formulation

In order to understand the effect of process parameters on the formulation, various parameters like the pH of coacervation medium, amount of cross linking agent and rate of addition of ethanol were varied. Our results revealed that the size of nanoparticles was inversely related to the pH of the medium characterized by a decrease in size from 700 to 150 nm for pH 4.0–9.0 respectively (Fig S1). This observation was in good agreement with the earlier work of Lin et al. [17] which suggests that a decrease in size of the nanoparticles could be due to increased ionization of BSA.

The glutaraldehyde cross linking procedure was identified as a crucial parameter for biodegradability and drug release of the nanoparticles. Hence the glutaraldehyde concentration was varied from 4% to 25%. On adding 4% of glutaraldehyde, the cross linking of the amino acid residues was minimal and therefore the nanoparticle yield was less. On increasing this to 25%, the formulation yielded micron sized particles. But when 100 µL of 8% glutaraldehyde were added, the average hydrodynamic size of particles were 125 nm and cross linking was found to be sufficient for the linkage of all the amino moieties in the lysine group of the particles (Fig S1) [18]. Similarly, the rate of addition of ethanol had great influence on the particle size distribution. Stable nanoparticles were obtained when the rate of addition of solvents were particularly high. The rapid desolvation process lead to the formation of large sized particles and this rate had to be optimized. Uniform distribution was seen when the rate of addition of ethanol was about 0.8 mL/min.

### Surface morphology, hydrodynamic diameter and zeta potential

The as synthesized ATV-BSA nanoparticles were highly stable in both water and cell media with an average hydrodynamic size in the range of 97–125 nm and polydispersity index between 0.29–0.40. Interestingly the ATV-BSA nanoparticles showed an increased hydrodynamic diameter (361 nm) comparatively higher than bare nanoparticles thus enumerating the entrapment of the drug. The stability of the nanoparticles can be attributed to the significantly higher zeta potential. Negatively charged particles contribute to the high stability of the colloidal solution. Coulombic repulsive forces between the particles prevent them from agglomerating in the colloid state thus maintaining the stability of the system [19]. The zeta potential value of the ATV-BSA nanoparticles was −36.3 mV which is high thus contributing for a stable system.

**Figure 1:**
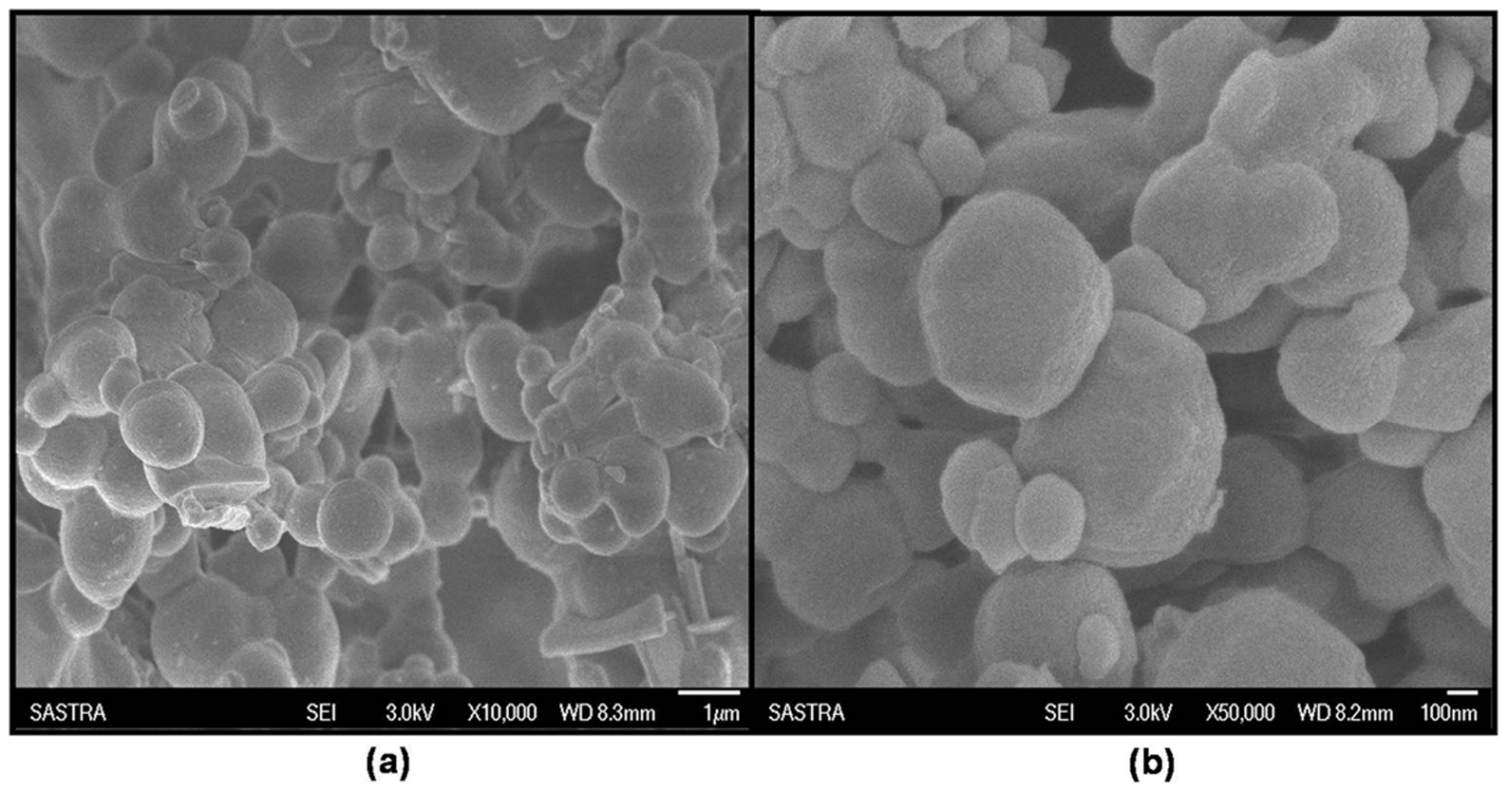
Scanning Electron Microscopic images. Field Emisssion Scanning Electron Microscopic images of (a) BSA nanoparticles (b) ATV-BSA nanoparticles.

The surface morphology of the particles was determined by the Scanning Electron Microscopy (SEM). The images of the BSA nanoparticles and ATV-BSA nanoparticles revealed a spherical morphology of the particles as shown in Fig. 1. Majority of the particles showed uniform size distribution without any crystal precipitation.

### FTIR spectroscopy

**Figure 2:**
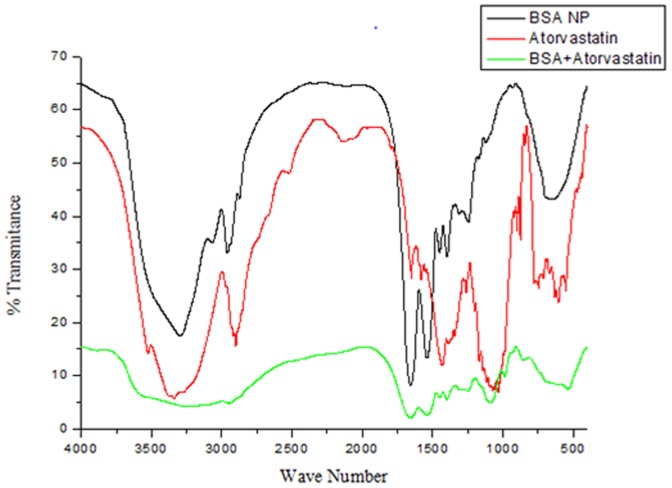
FTIR spectra. Shows the Fourier Transform Infra Red Spectra of (a) BSA nanoparticles (b) ATV and (c) ATV-BSA nanoparticles in order of their arrangement from top to bottom.

FTIR analysis helped in understanding the chemical modifications that occurred to the nanoparticles following drug interaction. Fig. 2 shows the FTIR spectra of BSA nanoparticles, ATV-BSA nanoparticles and ATV. The characteristic peaks of atorvastatin were recorded at 3364.21 cm^−1^ and 1649.81 cm^−1^ indicating aromatic N-H stretching and C = O stretching respectively, whereas the characteristic bands present in protein nanoparticles are Amide I at 1650 cm^−1^, Amide II band at 1530 cm^−1^ and Amide III region at 1230 cm^−1^. The ATV-BSA nanoparticle spectra were marked by the broadening of the N-H peaks indicating the possible interaction of the aromatic residues (tryptophan, tyrosine) of BSA with the drug.

### Fluorescence studies

**Figure 3:**
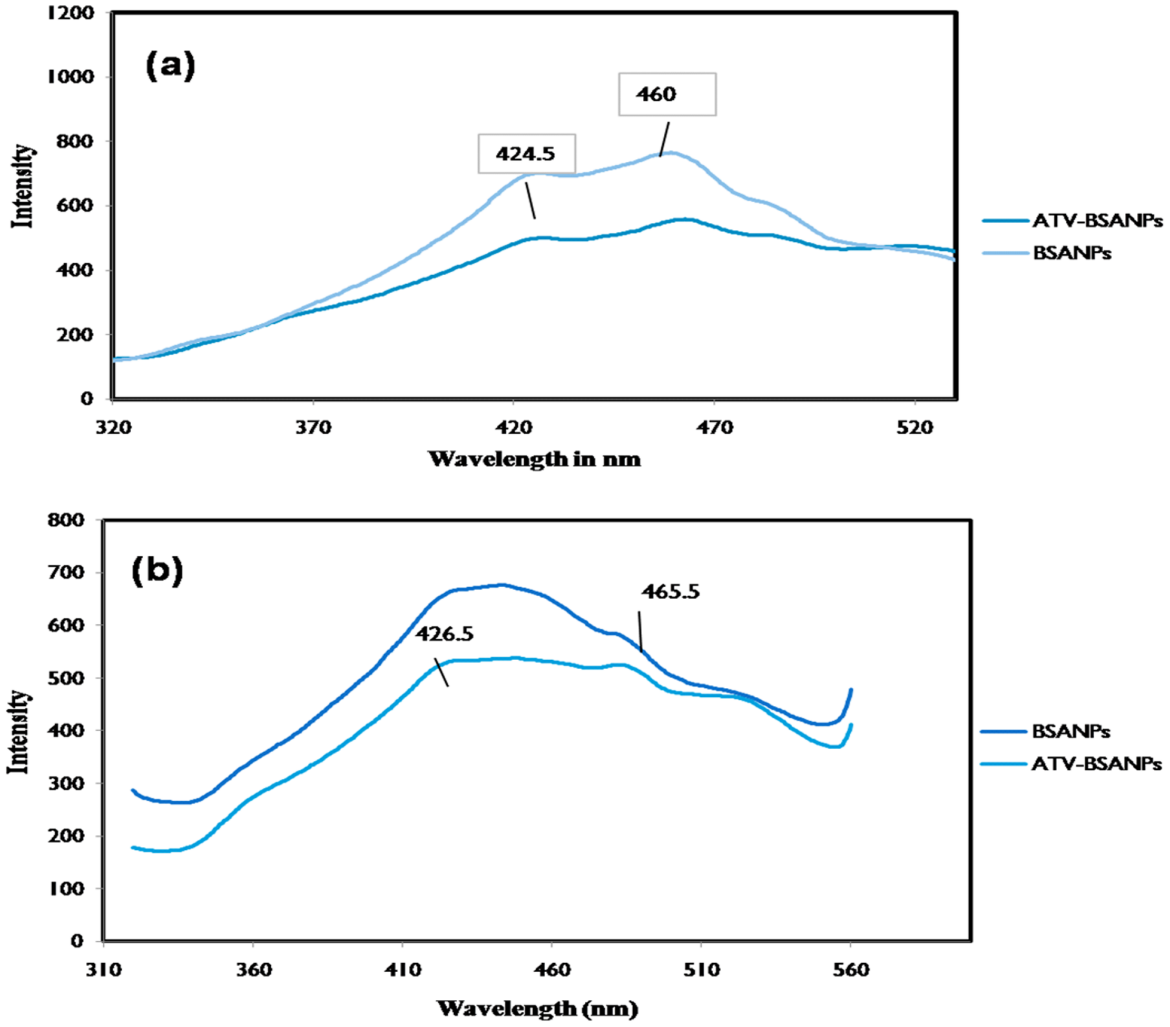
Fluorescent spectroscopy. The fluorescence emission spectra of BSA and ATV- BSA nanoparticles following excitation at (a) 293 nm (b) 280 nm respectively.

Further in order to confirm the nature of interaction of ATV to BSA and to corroborate the results obtained in FTIR spectroscopy, fluorescence spectroscopy was carried out. It also gives a clear picture on the internal environment around the vicinity of the fluorophore. Upon excitation at 293 nm, the tryptophan residues in BSA displayed a strong decrease in the intensity in the spectra of ATV-BSA nanoparticles in comparison with the bare nanoparticles (Fig. 3a). This decrease in the intensity could be due to fluorescence quenching attributed to the changes in the microenvironment of the tryptophan residues suggesting interaction of ATV and BSA. The quenching process also signifies the tertiary structural changes in BSA [20]. The hydrophobic binding pocket in the sub domain IIA would have undergone conformational changes with the possible interaction of BSA and ATV or overexposure of the residue to water [21]. The same quenching effects were noticed in Fig. 3b when the ATV-BSA nanoparticles were excited at 280 nm. This excitation wavelength corresponded to the tryptophan and tyrosine residues of the test sample. Hence the results obtained from FTIR and Fluorescence spectroscopy clearly indicate the major involvement of aromatic residues in the drug carrier interactions.

### Drug release profile

**Figure 4:**
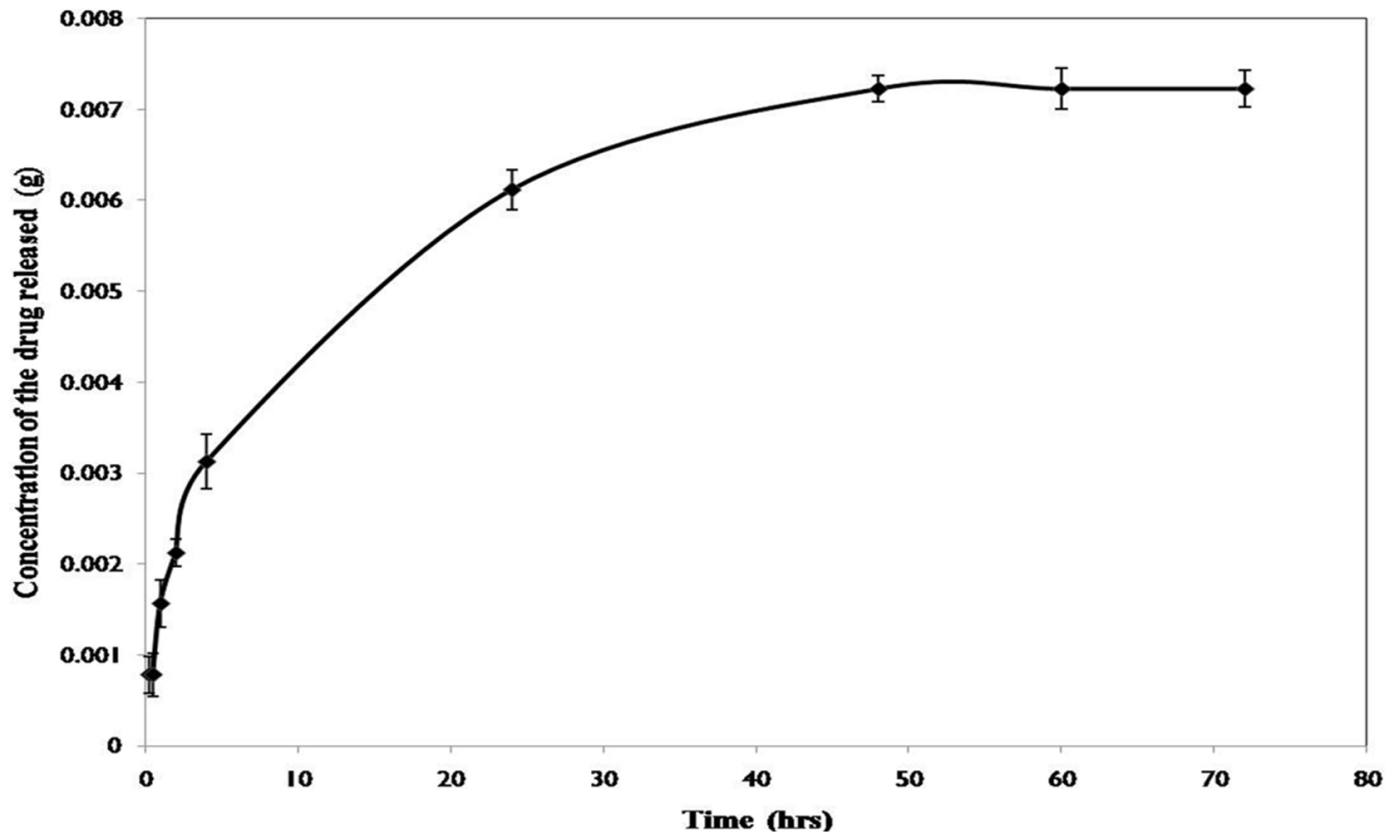
ATV release kinetics. *In vitro* drug release profile at pH 7.4 showing the sustained release of ATV from the nanoparticles at pre defined time intervals.

We investigated the *in vitro* release profile of ATV from the nanoparticles at pre defined time intervals at pH 7.4. A biphasic drug release pattern was seen with the initial burst release for 2 hours followed by a controlled release of the drug. The plateau region seen after few hours corresponded to the sustained release of the drug making it an effective carrier. As shown in Fig. 4, about 85% of the drug was released in 72 hrs at specified intervals.

The cross linking of the BSA nano carriers using glutaraldehyde was the major reason for the enhanced stability of the particles in PBS. Our results revealed that slow release of the drug in the media makes it an ideal carrier for drug delivery.

### Encapsulation efficiency

Various combinations of the drug with the nanoparticle were taken and synthesis was done using the desolvation method. The supernatant after centrifugation was analysed to determine the amount of free drug. The encapsulation efficiency was found using the formula
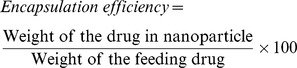



The drug: BSA combination with the best encapsulation efficiency was taken for further studies. As shown in Table 1, 10∶20 ratio of drug: BSA gave the best encapsulation efficiency of 94±0.32%.

**Table 1:** Influence of different ratios of ATV to BSA (20 mg) on Encapsulation Efficiency.

Sl No	Drug concentration (mg)	Encapsulation efficiency
1.	5	71±0.45%
2.	10	94±0.32%
3.	15	91±0.87%
4.	20	73.33±0.68%

### Haemolysis assay

**Figure 5:**
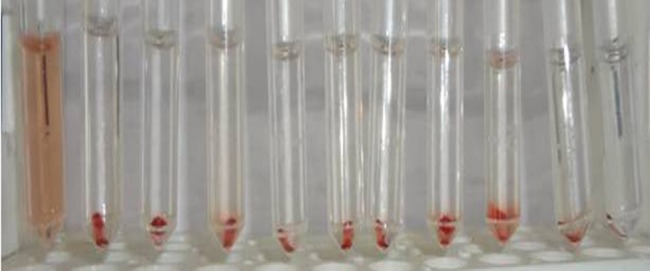
Hemolysis assay. The biocompatibility of ATV-BSA nanoparticles is shown with tubes from left to right as: PC-Positive control, NC-Negative Control, a to c, d to f and g to i showing the biocompatibility of 10,15 & 20 µg/mL concentrations of ATV, BSA and ATV-BSA nanoparticles in RBC's respectively.

The results of the haemolysis assay can be analysed from Figure 5. Three different concentrations of ATV alone, BSA nanoparticles, ATV-BSA nanoparticles were incubated with the blood samples and haemocompatibility effects were tested. Our results revealed that all the above tested concentrations neither exhibited haemolytic activity nor thrombus formation making it suitable for circulation in the blood. No adverse reactions could be observed between the serum proteins and the surface of the nanocarrier. Distilled water which was used as a positive control exhibited 100% haemolysis marked by complete lysis of the Red Blood Cells (RBC's) as shown in Fig. 5. Whereas saline which was used as the negative control, bare drug and nanoformulation did not show any haemolysis or toxicity to the RBC's making it a clinically promising formulation. Our results were consistent with results obtained by Wang et al, 2010 which discusses the haemocompatibility of docetaxel ­ loaded albumin nanoparticles [22].

### Cytotoxicity assays

#### Determination of ROS content

**Figure 6:**
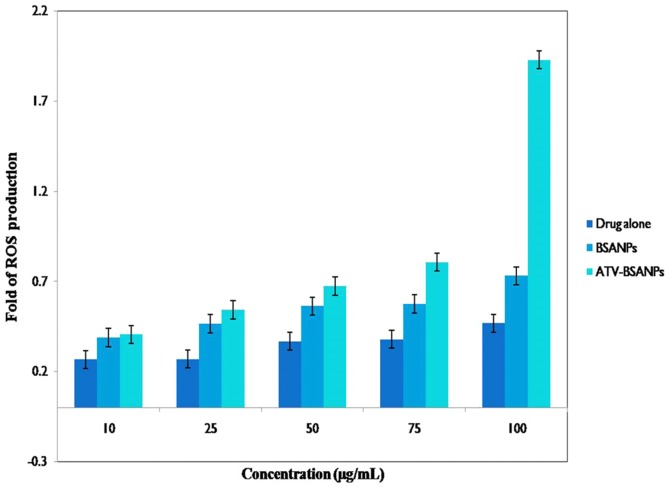
ROS Assay. Shows the effect of free radicals on the viability of MiaPaCa-2 cells upon treatment with ATV, ATV-BSA and carrier (BSA). The error bars represents standard deviation of quadruplicates.

The cytotoxicity effects might have occurred through the induction of oxidative stress and apoptosis with possible involvement of overproduction of reactive oxygen species (ROS). In this regard, the ability of ATV-BSA nanoparticles to induce intra cellular oxidant production in MiaPaCa-2 cell lines was assessed by measuring DCF fluorescence as an indicator of ROS generation (Fig. 6). Various concentrations (10, 25, 50, 75, 100 µg/mL) of ATV-BSA and ATV were used for the study. The bare drug exhibited a concentration dependent production of ROS, but the results were not significant when compared to the ATV-BSA nanoparticles. Interestingly, 10, 25, 50 µg/mL of ATV-BSA did not induce significant generation of ROS. Whereas as the concentration of the nanoparticles increased further to 75 and 100 µg/mL a remarkable ROS production is seen. Our results revealed that though ROS production is not the primary cause for cytotoxicity, a significant amount contributes to the cellular damage.

#### Determination of cell viability

**Figure 7:**
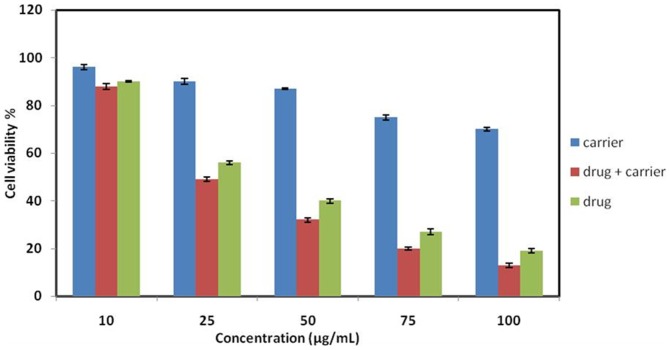
MTS Assay. Cytotoxicity of BSA nanoparticles, ATV & ATV-BSA nanoparticles at equal concentrations as measured on MiaPaCa-2 cells using MTS assay.

The anticancer activities of the ATV–BSA nanoparticles against MiaPaCa-2 cell lines have been investigated for future potential clinical applications. As depicted in Fig. 7, the inhibition effect has been observed when MiaPaCa-2 cells were incubated with ATV–BSA nanoparticles, indicating the presence of anticancer activities. Besides, the inhibition effect increased with the desired concentration of ATV increasing from 10 to 100 µg/mL. The enhanced effect of inhibition could be attributed to the uptake of ATV–BSA. It should be taken into account that bare ATV also exhibited toxicity, but the extent of toxicity was less when compared to ATV-BSA nanoparticles. A student T test was performed and the values obtained were statistically significant (p<0.05). In addition, the BSA nanoparticles without ATV were incubated with MiaPaCa-2 cells as control experiments, and little cytotoxicity to cells was observed with the increase in concentration, which was calculated according to the drug loading amount compared with the ATV-BSA nanoparticles.

#### Cell uptake studies

**Figure 8:**
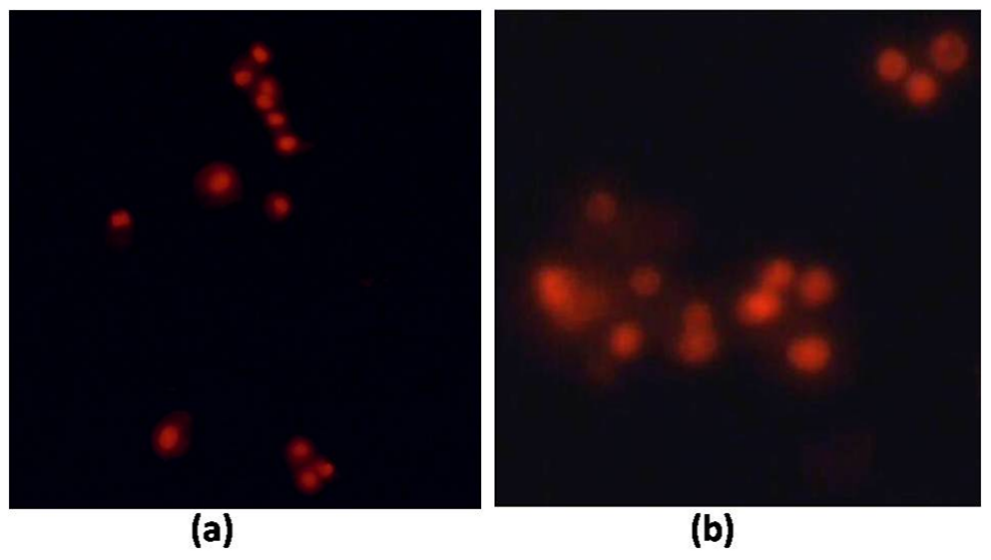
Propidium Iodide Staining. Shows the dead cells stained red using propidium iodide upon treatment with ATV and ATV-BSA on MiaPaCa-2 cells.

**Figure 9:**
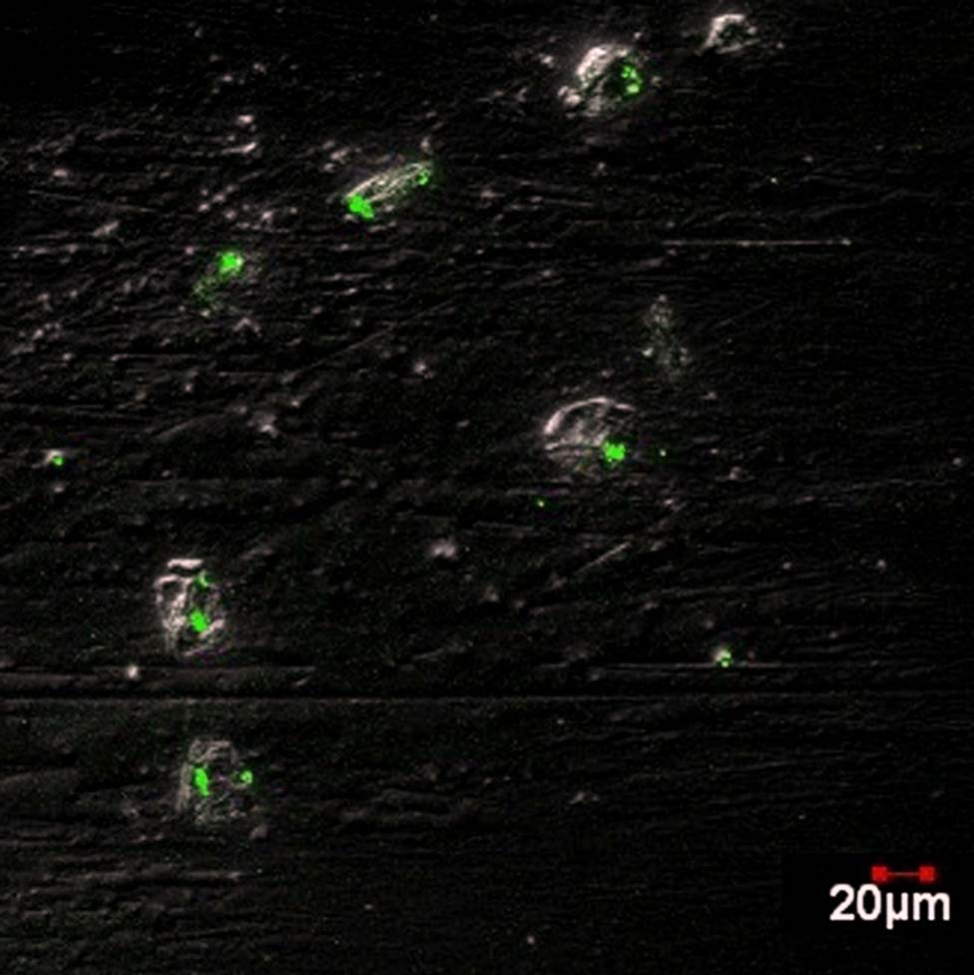
Confocal Microscopy. Shows the cellular uptake of ATV-BSA nanoparticles by MiaPaCa-2 cells using confocal microscopy.

The cellular uptake of the ATV-BSA nanoparticles and ATV was monitored in vitro by staining the cells with propidium iodide (PI). PI selectively binds to the nucleus of the dead cells as shown in Fig. 8. Our results revealed that following incubation at 37°C, the cells incubated with ATV-BSA were marked by a significant induction of cell death characterized by distortions in cellular morphology of the cells when compared to the bare drug at various time intervals. Confocal imaging of the cells following FITC-loaded ATV-BSA nanoparticle delivery to MiaPaCa-2 cells showed uniform distribution of the fluorescent dye to nucleus thus confirming efficient delivery of the cargo to the cells (Fig. 9). The mechanism of toxicity however is not completely clear, and it is possible that more than one mechanism is involved.

According to previous reports generation of oxidative stress is considered to be a key mechanism involved in cellular toxicity [23], [24] but studies revealed that free radicals may not be a major contributor to toxicity. In this regard, we suggest that caveolin mediated endocytosis could be another probable mechanism of cellular uptake and toxicity. Since most of the experiments using nanoparticles of varying size and composition determined that uptake of nanoparticles depends strongly on their size and shape [25], ATV-BSA nanoparticles can easily be taken up by the cells. For example a previous report on curcumin loaded albumin nanoparticles suggests that the successful endocytic cellular uptake along with successful tumor retention of albumin nanoparticles leads to a successful passive targeting of drugs to tumors with this delivery system [26].

**Figure 10:**
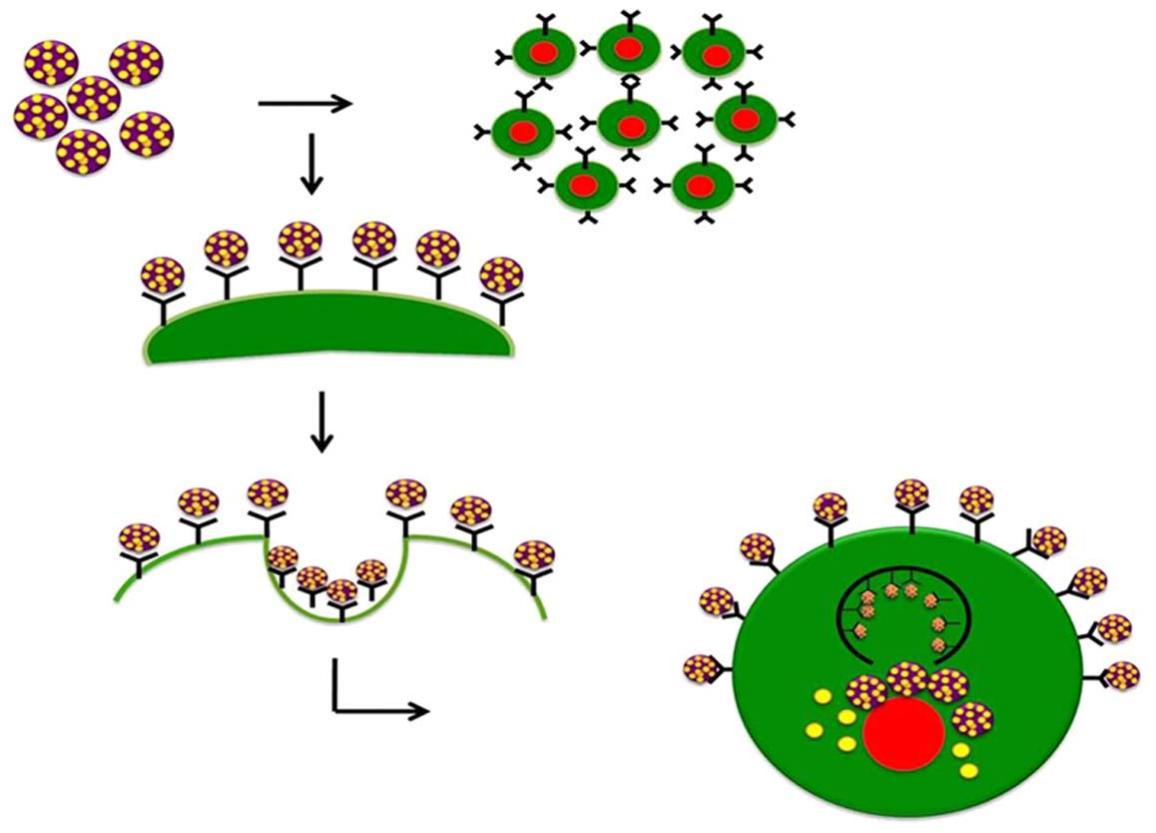
Possible Mechanism. Schema representing the caveolae mediated endocytosis as possible mechanism involved in the cellular uptake of the ATV-BSA by the cancer cells.

As shown in Fig. 10 nanoparticles adhere to the cell surface through specific interactions via reversible ligand–receptor binding. Upon attaining thermodynamic equilibrium, the NPs are wrapped by the cell membrane and endocytosed. Our hypothesis is supported by the results obtained by Rejman et al which suggest that particles of at least 200 nm and less than 1 µm in diameter are rapidly taken up by the cells preferentially along the pathway of caveolae-mediated endocytosis [27]. A few related studies have also reported that albumin is capable of binding to the gp60 receptor which triggers its binding to an intracellular protein (caveolin-1) resulting in subsequent invagination of the cell membrane to form transcytotic vesicles, i.e. caveolae [28].

### Molecular Docking

**Figure 11:**
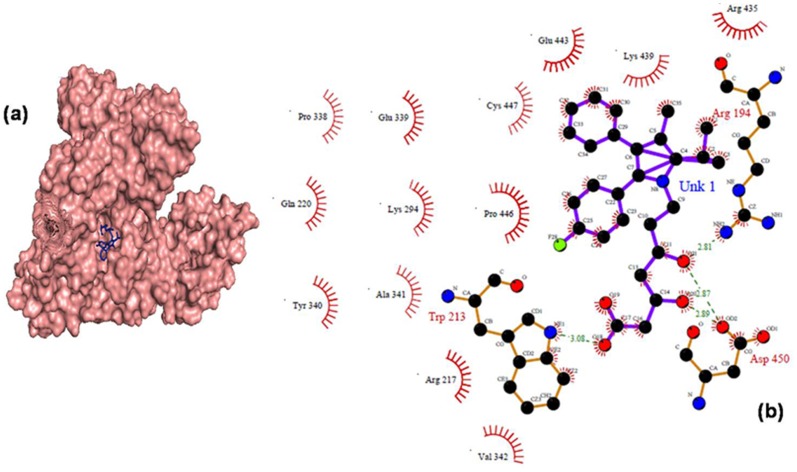
Molecular Docking. Graphical representation of best docking results of BSA-atorvastatin complex using Autodock 4.2. Residual interactions at the BSA-ATV interface in BSA. The symbol coding scheme is as follows. BSA in green color, ATV in blue color. Hydrogen bonding interactions are denoted by dashed lines. Residues involved in the hydrophobic interactions are shown as starbursts.

Among the various conformers of docking results, only top 3 conformers were taken on the basis of the free energy of binding and score ranking. The minimum binding energy conformer is shown in Fig. 11 and all the related information are given in Table 2. Based on a previous report, there are two drug binging grooves located in BSA, namely site I and site II, which are present in the hydrophobic cavities of sub domain IIA and IIIA [29]–[31]. In the minimum energy conformer, atorvastatin binds within the sub domain IIA pocket, forming hydrogen bonding with Trp- 213(3.08 Å) as shown in Figure 11. Additionally, a number of hydrophobic and electrostatic interactions were formed from the ionic and polar groups around the drug molecule. From the docking simulation, the observed binding energy for ATV-BSA nanoparticles was – 4.35 kcal/mol. Our observations were consistent with the findings by Chakrabarti et al. [32], and Jana et al. [33] in which Trp 213 plays a crucial role in drug interaction. Docking results showed stronger drug-protein complex formed with BSA which is consistent with our fluorescent results.

**Table 2:** Docking analysis of BSA with ATV showing the lowest binding energy conformers in kcal/mol.

Rank	Hydrogen bonds (Å)	Binding energy (kcal/mol)	Reference RMS
1	Trp −213, Asp – 450	−4.35	8.26
2	Trp −213	−4.01	10.02
3	Trp 213	−3.99	9.35

## Conclusion

The manuscript discusses about the anticancer properties of ATV loaded BSA nanoparticles with minimal particle size and maximum drug loading efficiency. Our results revealed that stable formulations of ATV-BSA can be obtained at pH 8, 0.8 mL/min addition of ethanol and 8% glutaraldehyde concentration. The *in vitro* studies revealed that ATV-BSA nanoparticles were biocompatible and showed noticeable cytotoxicity to MiaPaCa 2 cells when compared to the bare drug. The successful encapsulation of the drug in the carrier enabled cellular uptake and cytotoxicity. In fact, a nanoparticle formulation of paclitaxel in which serum albumin is included as a carrier (Abraxane) has been approved for the treatment of breast cancer. Hence our studies suggest that the chemotherapeutic properties of ATV can be better exploited by encapsulating it in a carrier for medical applications.

## Supporting Information

Figure S1 **Optimization of the process parameters for the formulation of BSA nanoparticles.**
(DOC)
