# Supplementary material for: BSA Nanoparticle Loaded Atorvastatin Calcium - A New Facet for an Old Drug
Source: PLoS One. 2014 Feb 3;9(2):e86317. doi: 10.1371/journal.pone.0086317 (PMC3911917; doi:10.1371/journal.pone.0086317)
Supplement: Figure S1 — Optimization of the process parameters for the formulation of BSA nanoparticles. (DOC) [file pone.0086317.s001.doc]

**Supplementary Information**

BSA Nanoparticle loaded Atorvastatin calcium-a new facet to an old drug

Sripriyalakshmi.S ^a^, Anjali. C.H ^a^, George Priya Doss. C ^b^, Rajith B ^b^, Aswathy Ravindran ^a §^

^a§^ Center for Nanotechnology and Advanced Biomaterials (CeNTAB), School of Chemical and Biotechnology, SASTRA University, Tirumalaisamudram, Thanjavur - 613401, India

^b^Medical Biotechnology Division, School of Biosciences and Technology, VIT University, Vellore - 632014, India

**Figure S1**

**Optimization of the process parameters for the formulation of BSA nanoparticles**

**
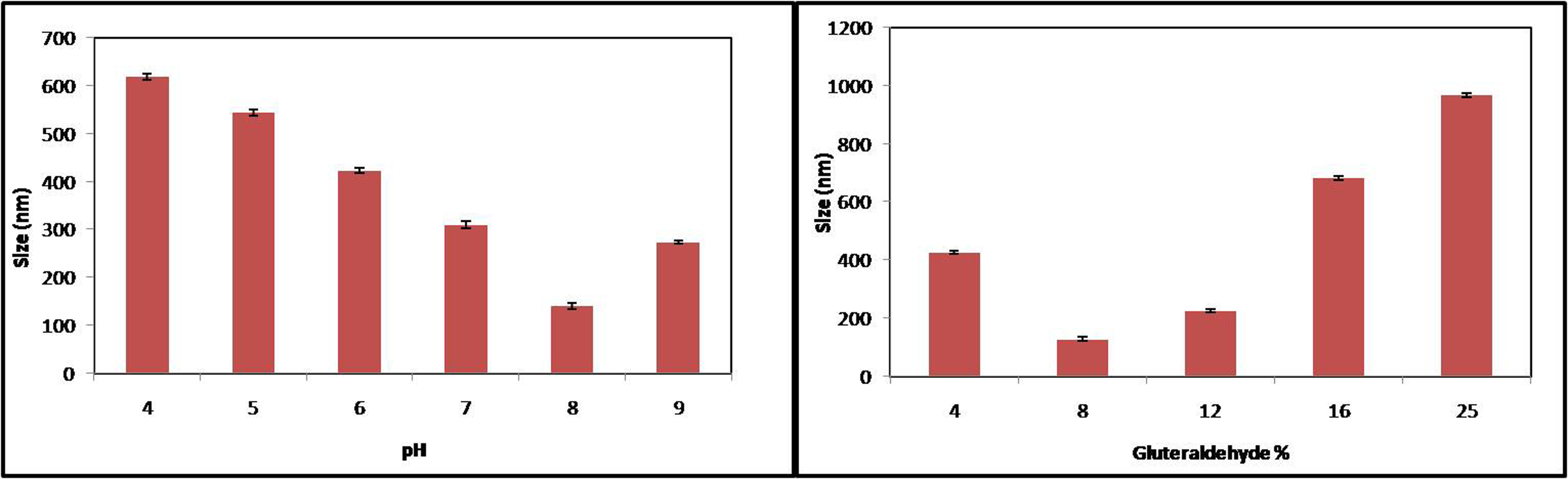
**
